# Supplementary material for: Textural and Consumer-Aided Characterisation and Acceptability of a Hybrid Meat and Plant-Based Burger Patty
Source: Foods. 2023 Jun 1;12(11):2246. doi: 10.3390/foods12112246 (PMC10253062; doi:10.3390/foods12112246)
Supplement: Supplementary file 1 [file foods-12-02246-s001.zip › foods-2403021-supplementary.pdf]

Table S1. Estimated nutritional content[(a)] per 100 g of a hybrid (50%/50% plant/meat) and a beef (100% meat) burger patty.

	**Amount**		
	**Hybrid**	**Beef**	**Unit**
Energy	772/185	789/189	kJ/kcal
Fat	10.5	12.0	g
Saturated fat	4.8	4.9	g
Carbohydrate	3.5	0.0	g
Sugars	0.3	0.0	g
Dietary fibre	1.0	0.0	g
Protein	19.0	20.0	g
Salt	1.3	1.5	g

[a)] Data used for nutritional calculations are described in the text.
